# Evaluation of tumor regression by neoadjuvant chemotherapy regimens for esophageal adenocarcinoma: a systematic review and meta-analysis

**DOI:** 10.1093/dote/doac058

**Published:** 2022-09-24

**Authors:** Swathikan Chidambaram, Viknesh Sounderajah, Nick Maynard, Richard Owen, Sheraz R Markar

**Affiliations:** Department of Surgery and Cancer, Imperial College London, London, UK; Department of Surgery and Cancer, Imperial College London, London, UK; Department of Molecular Medicine and Surgery, Karolinska Institutet, Stockholm, Sweden; Department of Molecular Medicine and Surgery, Karolinska Institutet, Stockholm, Sweden; Department of Molecular Medicine and Surgery, Karolinska Institutet, Stockholm, Sweden; Department of Surgery, Churchill Hospital, Oxford University Hospitals NHS Trust, Oxford, UK

**Keywords:** esophageal adenocarcinoma, chemoradiotherapy, neoadjuvant chemotherapy, pathological complete response

## Abstract

Locally advanced esophageal adenocarcinomas (EACs) are treated with multimodal therapy, namely surgery, neoadjuvant chemotherapy (NAC) or chemoradiotherapy (CRT) depending on patient and tumor level factors. Yet, there is little consensus on choice of the optimum systemic therapy. To compare the pathological complete response (pCR) after FLOT, non-FLOT-based chemotherapy and chemoradiotherapy regimes in patients with EACs. A systematic review of the literature was performed using MEDLINE, EMBASE, the Cochrane Review and Scopus databases. Studies were included if they had investigated the use of chemo(radio)therapy regimens in the neoadjuvant setting for EAC and reported the pCR rates. A meta-analysis of proportions was performed to compare the pooled pCR rates between FLOT, non-FLOT and CRT cohorts. We included 22 studies that described tumor regression post-NAC. Altogether, 1,056 patients had undergone FLOT or DCF regimes, while 1,610 patients had received ECF or ECX. The pCR rates ranged from 3.3% to 54% for FLOT regimes, while pCR ranged between 0% and 31% for ECF/ECX protocols. Pooled random-effects meta-meta-analysis of proportions showed a statistically significant higher incidence of pCR in FLOT-based chemotherapy at 0.148 (95%CI: 0.080, 0.259) compared with non-FLOT-based chemotherapy at 0.074 (95%CI: 0.042, 0.129). However, pCR rates were significantly highest at 0.250 (95%CI: 0.202, 0.306) for CRT. The use of enhanced FLOT-based regimens have improved the pCR rates for chemotherapeutic regimes but still falls short of pathological outcomes from CRT. Further work can characterize clinical responses to neoadjuvant therapy and determine whether an organ-preservation strategy is feasible.

## INTRODUCTION

Esophago-gastric (EG) cancers are aggressive malignancies that were once associated with a poor prognosis, partly due to their primary management with surgery alone.^1^^,^^2^ In the past two decades, the centralization of oncological services, introduction of minimally invasive surgery, the incorporation of systemic primary oncological therapies and importantly better understanding of tumor biology have led to improvements in the survival.^3^ In fact, between 1990 and 2017, the age-standardized mortality decreased by 29%, and disability-adjusted life years (DALYs) decreased by 33.4% globally.^4^ Currently, locally advanced EG cancers are treated with multimodal therapy, namely surgery, chemotherapy and/or chemoradiotherapy (CRT) depending on patient and tumor level factors. The specific regime has been the subject of several trials, leading to evolution in oncological practice. These include the MAGIC, CROSS and more recently the FLOT4 trials. In 2006, the MAGIC trial showed that a perioperative regimen of ECF (epirubicin, cisplatin and fluorouracil) decreased tumor size and stage and significantly improved progression-free and overall survival (OS) of patients with operable OG cancers.^5^ In 2012, the CROSS trial demonstrated the effectiveness of radiotherapy alongside carboplatin-paclitaxel based regime, which substantially improved OS compared with surgery alone.^6^

The exact protocol of chemotherapy or CRT may vary between centers; however, they converge in their aim to downstage the tumor toward complete regression before surgical intervention is carried out.^7^ This is pertinent given the abundant evidence supporting that a pathological complete response (pCR) is strongly associated with better survival outcomes.^8^^,^^9^ However, there is considerable difference between the effectiveness of regimes. For example, while the recurrence free survival (RFS) is 75% in patients who underwent CRT, the 5-year RFS following a pCR in resected patients who underwent chemotherapy is significantly higher at 87%. This may be attributed to the introduction and uptake of taxane-based regimes as such as the FLOT (fluorouracil plus leucovorin, oxaliplatin and docetaxel) protocol that has come to the forefront as a commonly used regime in most centers in the United Kingdom (UK). In fact, for patients with locally advanced, resectable gastric or gastro-esophageal (GEJ) junction adenocarcinomas, the FLOT trial demonstrated a significantly higher OS compared with perioperative ECF/ECX regimes.^10^ However, there is no other work comparing or summarizing the pCR to the various regimes used in current practice. Thus, this systematic review aims to compare the pCR to FLOT and non-FLOT-based chemotherapy regimes as well as chemoradiotherapy (CRT) protocols in patients with esophageal adenocarcinoma.

## METHODS

Literature search methods, inclusion and exclusion criteria, outcome measures and statistical analysis were defined according to the Preferred Reporting Items for Systematic Reviews and Meta-Analyses (PRISMA).^11^ Patients were not involved in the conception, design, analysis, drafting, interpretation or revision of this research. Hence, ethical approval was not required and thus not sought for this study.

### Literature search

The following databases were searched: MEDLINE (1946 until the first week of February 2022) via OvidSP; MEDLINE in-process and other non-indexed citations (latest issue) via OvidSP; Ovid EMBASE (1974 to latest issue) and Scopus (1996 till present). The last search was performed on February 2022. Two separate searches were performed to include all articles that evaluated various oncological therapies for esophageal adenocarcinoma. Search terms used several strings which were linked by standard modifiers in the following order: ‘oesophageal cancer’, ‘esophageal cancer’, ‘esophageal adenocarcinoma’, ‘oesophageal adenocarcinoma, OAC’ OR ‘EAC’; ‘chemotherapy’, ‘neoadjuvant’ OR ‘NAC’ and ‘chemoradiotherapy’, ‘radiotherapy’ OR ‘CRT’. The strings were then combined using the AND modifier. References of included articles were screened and a hand-search was performed to identify missing articles. Two reviewers (S.C. and V.S.) independently assessed the titles and abstracts for inclusion of relevant references. In cases where there was disagreement for inclusion, a third author (R.O.) was consulted.

### Selection of studies

Studies were included if they had investigated the use of chemo(radio)therapy regimens in the neoadjuvant setting for management of patients with esophageal adenocarcinoma. Studies were only included only if they reported the rate of pCR. Study design was restricted to only randomized clinical trials (RCTs) and cohort studies. Studies were excluded if they did not investigate neoadjuvant chemo(radio)therapy; did not report the pCR rates; had mixed cohorts of esophageal cancer histological subtypes; patients underwent only adjuvant chemotherapy; had incomplete data on outcome measures or not in the English language. Studies with incompatible designs including case series, letters, comments and reviews were also excluded.

### Outcome measures and data extraction

Our main aim was to assess the impact of neoadjuvant chemotherapy (NAC) on the regression of tumor prior to surgery, hence the pCR rate was selected to be the primary outcome measure. In addition, the following data were extracted from each study: first author, year of publication, study design, sample size, demographic data (age and gender), oncological details (pathological stage); surgical intervention (operation technique or approach) and details of chemotherapy agents and radiotherapy regimes used.

### Quality assessment of selected studies

Two reviewers (S.C. and S.R.M.) assessed the quality of each included study by independently evaluating the risk of bias using the Newcastle–Ottawa Scale (NOS) for the assessment of non-randomized studies.^12^ The NOS scores ranging from 0 to 9, with a higher score indicating a lower risk of bias. In this review, we considered a score of 0 to 3, 4 to 6 and 7 to 9 as low, moderate and high quality of studies, respectively.

### Statistical methods

Review Manager 5.3 (Cochrane Collaboration, Oxford, UK) was used for statistical analysis of the data. Two types of modeling were used to assess the heterogeneity of the data: fixed-effects and random-effects. The random-effects model was chosen for all analysis due to the significant heterogeneity between studies. Data are given as odds ratio and 95% confidence intervals (CIs) for all non-continuous data, and as standardized mean difference and 95% CI for all continuous data. In all cases, statistical heterogeneity was assessed by using *I*^2^ statistic and was categorized as low, moderate and high for an *I*^2^ statistic of above 25%, 50% and 75%, respectively. Results above 60% were considered as substantial heterogeneity.

## RESULTS

### Study selection

**Fig. 1:**
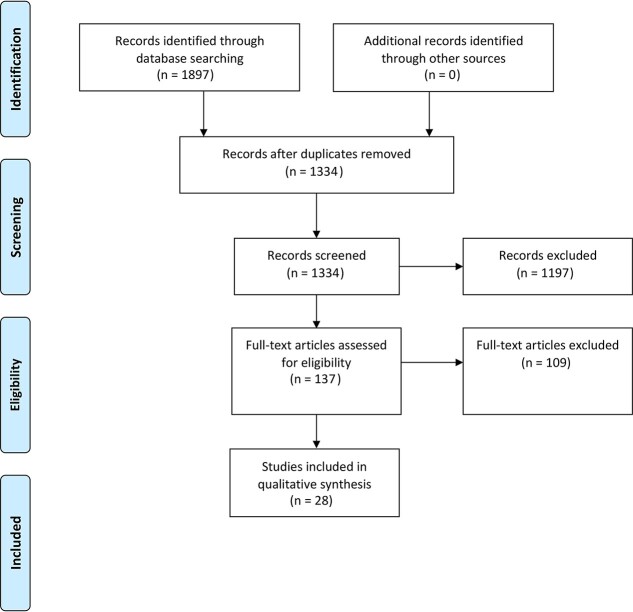
PRISMA flow diagram.

The database search yielded a total of 1,897 studies. After duplicates were removed, titles and abstracts of the remaining 1,334 studies were assessed for eligibility, and 1,197 studies were removed. A further 109 studies were excluded after full-text review due to incompatible outcome measures or study design (Fig. 1).^13^ Twenty-nine studies that reported pCR as a measure of tumor regression post-NAC or CRT were included (Table 1).

**Table 1:** Characteristics of included studies

**Study**	**Design; Arms**	**Sample size**	**Sex (% males)**	**Age**	**Pathologic TNM stage**	**Chemotherapy** **regime**	**Operative characteristics**	**PCR**	**OS**	**pCR vs. no pCR OS**
Donlon 2021	RC	175	84	66 (28–83)	T0 16 (10%) T1 27 (17%) T2 23 (14%) T3 86 (53%) T4 9 (6%)	FLOT	Open 123 (76%) Hybrid 19 (12%) MIE 19 (12%)	PCR: 10% Major response: 24%	1-year: 91% 3-year: 60%.	—
Hoepner 2014	RC	46	92	62 (31–77)	T0 20 (19%) T1 14 (12%) T2 36 (35%) T3 34 (33%) T4 1 (0.9%)	Before 2010: ECF After 2010: FLOT	Open, thoraco-abdominal (95%) Open, Transhiatal (5%)	Total/subtotal: 30% Minimal/none: 55%	5-year: 45%	pCR: 30/48 npCR: 24/57
Lorenzen 2012	RC	120	80	59.5	T0 18 (15%) T1/2 77 (64%) T3/4 25 (21%)	Docetaxel, Cisplatin Folinic acid	Not given	15%	3-year: 71.8% (PCR) and 37.7% (non-PCR)	pCR: 10/18 npCR: 39/102
Thuss-Patience	RCT	51	94.1	65 (37–74)	Not given	DCF	Not stated	13.7	1 year: 88 2 year: 70.8	pCR: 2/7 npCR: 16/44
Misra 2012	RC	99	92	<50: 9 50–80: 92 >80: 9	T0 19 (%) T1 18 (%) T2 27 (%) T3 41 (%) T4 5 (%)	Cisplatin 5-FUDR, docetaxel, leucovorin	Open	38%		
Luc 2015	RC	61	83.6	64.5 (40–78)	T0 (%) T1 (%) T2 (%) T3 (%) T4 (%)	Docetaxel, cisplatin, 5-FU	Open, Ivor-Lewis	3.3%	Median of 45 months	
Port 2007	RC	51	84	62 (36–76)	T0 4(7%) T1 3 (5%) T2 11 (18%) T3 22 (36%) T4 12 (20%)	Paclitaxel, Carboplatin	Not mentioned	pCR: 4.8% Major response: 11.2%	5 year: 35%	
Lim 2013	RC	83	79.5	62 (37–79)	Not mentioned	ECF	Not mentioned	7.69%	Median: 38 months	pCR: 6/6 npCR: 38/77
Starling 2009	RCT	34	91	60 (41–81)	T2 7 (21%) T3 17 (79%)	Epirubicin, cisplatin, capecitabine	Not mentioned	7.4%	Median: 17 months 1 year: 67% 2 year: 39%	pCR: 2/2 npCR:12/32
Alderson 2017	RCT (OEO5)	897	90.3	62 (27–81)	T0 25 (3.36%) T1 77 (10.3%) T2 125 (16.8%) T3 493 (66.4%) T4 22 (2.96%)	Cisplatin, fluorouracil: 339 Epirubicin, cisplatin, capecitabine: 317	Open: 379 Left thoracoabdominal: 52 Hybrid: 209 MIE: 18	3.96%	Median for CF: 23.4 months Median for ECX: 26 1 months	pCR: 25/41 npCR: 128/543
Schuhmacher 2010	RCT	144	69.4	56 (38–70)	T0 5 (7.1%) T1 5 (7.1%) T2 36 (51.4%) T3 20 (28.6%) T4 4 (5.7%)	Cisplatin, Fluorouracil, Folinic acid	Open	7.1%	Stopped early for poor accrual	
Peixoto 2014	RC	83	79.5	62 (37–79)	T0 (%) T1 (%) T2 (%) T3 (%) T4 (%)	ECF: 23 Epirubicin, cisplatin, capecitabine: 60	Not mentioned	7.2%	Median: 40.3 months	pCR:6/6 npCR: 39/77
Geh 2000	RCT	23	78.2	54 (31–73)	T0 (%) T1 (%) T2 (%) T3 (%) T4 (%)	ECF	Open	4.35%	Median: 12 2 year: 30	pCR: 1/1 npCR: 7/22
Melcher	RCT	27		54 (29–71)	T0 (%) T1 (%) T2 (%) T3 (%) T4 (%)	ECF	Open	0%	Median: 14 2 year: 32	
Bamias	RCT	62		59 (28–79)	T0 (%) T1 (%) T2 (%) T3 (%) T4 (%)	ECF	Open	10%	Median: 12 2 year: 23	
Bradley 2019	RC	44	70.5	70 (35–85)	T2 1 (2.3%) T3 17 (38.6%) T4 26 (43.2%)	ECX/ECF	Total gastrectomy 8 Subtotal gastrectomy 2 Oesophagectomy 7	2.27%	Median: 42.6	
Mesenas 2008	RC	99	80	62	T0 8 (8%) T1 6 (6%) T2 22 (22%) T3 53 (54%) T4 10 (10%)	ECF	Open	8%	Median OS of 22 months	
Ott *et al.* 2011	RC	114	95	58.7 (10.3)	T0 12 (11%) T1 16 (13%) T2 31 (81%) T3 48 (29%) T4 1 (5%)	Cisplatin Folinic acid, leucovorin	Open; Transhiatal with cervical anastomosis, OR Abdominothoracic with intrathoracic anastomosis	31%	Median OS of 108 months	
Burmeister 2005	RCT	256	83	61 (41–80)	NA	35Gy/2.3Gy/15f Cisplatin, flurouracil	Thoraco-abdominal approach;	9.09 (6/66) %	Median OS 22.2 months	
Stahl *et al.*	RCT	101	91.5	NA	T3 108 T4 11	30Gy/2Gy/15f Fluorouracil, folinic acid, cisplatin, etoposide	Right transthoracic oesophagectomy; Extended total gastrectomy with resection of the lower oesophagus	Chemotherapy: 1.9% CRT: 14.3%	Chemo: OS 21 months; 5 years – 24.4% CRT: OS 30.8 months; 5 years—39.5%	
Burmeister *et al.* 2011	RCT	75	88	61 (36–75)	T2 67 T3 8	35Gy/2.3Gy/15f Cisplatin, fluorouracil	Thoracoscopically assisted three-stage dissection; Ivor-Lewis two-stage approach	Chemotherapy: 8% CRT: 31%	Chemo: 29 months; 5 years – 36% CRT: 32 months; 5 years—45%	
Shapiro *et al.*	RCT	368	75%	60 (55–67)	T1 1 T2 26 T3 150 T4 0	41.4Gy/1.8Gy/23f Carboplatin, paclitaxel	Transthoracic oesophageal resection; Transhiatal oesophageal resection	CRT: 29%	CRT: 48.6 months 5 year OS – 47%	pCR: 33/47 npCR: 57/113
Mariette *et al.*	RCT	195	85.6	57.8 (36.9–76.4)	T0 34 T1 49 T2 32 T3 43 T4 12	45Gy/1.8Gy/25f Cisplatin, fluorouracil	transthoracic esophagectomy	33.3%	Median – 31.8 months. 5 years: 41.1	pCR: 23/27 npCR: 19/49
Walsh et al	RCT	113	73.4	65 (47–75)	T0 13 T1 3 T2 35 T3 51 T4 8	30Gy/2Gy/15f Fluorouracil, folinic acid, cisplatin, etoposide	total gastrectomy and distal esophagectomy; Lewis–Tanner operation; McKeown operation; Transhiatal oesophageal resection	25%	Median: 16 months 3 year survival: 37%	pCR: 11/13 npCR: 15/32
Urba *et al.*	RCT	100	80	62 (39–75)	NA	45Gy/1.5Gy Cisplatin, fluorouracil, vinblastine	Transhiatal Esophagectomy	24.3%	Median: 16.9 3 year survival: 30%	pCR: 6/9 npCR: 5/28
Tepper *et al.*	RCT	475	93	59.9 (38–77)	NA	50.4 Gy/1.8 Gy Cisplatin, fluorouracil	Ivor-Lewis two-stage approach	40%	Median OS 4.48 years. 5 year survival: 39%	
von Dobeln	RCT	181	82.3	63 (37–75)	T1 2 T2 62 T3 117 T4	40 Gy/2 Gy/20f Cisplatin, fluorouracil	thoracoabdominal Ivor–Lewis resection; McKeown operation	CRT: 28% NAC: 9%	CRT: 42.2%, 31.4 months NAC: 39.6%, 36 months	
Kamarajah	RC	718	320 (87.0) 296 (84.6)	63.0 (9.9) 64.5 (9.5)	T1 120 T2 120 T3 231 T4 24	FLOT vs CROSS	Ivor Lewis, McKeown, thoracoabdominal, trans-hiatal	NAC: 10.1% CRT: 17.7%	—	
Klevebro	RCT	181	77 (85) 72 (80)	63 (37–75) 63 (38–74)	T1 2 T2 62 T3 117	Cisplatin, 5-FU, 40 Gy radiotherapy	Ivor Lewis, McKeown	NAC: 9% CRT:28%	—	

RCT: randomized controlled trial; RC: retrospective cohort study; CRT: chemoradiotherapy.

### Quality appraisal

Assessment of studies using the Newcastle–Ottawa tool showed that studies were of a moderate-high quality (Table **2**). The non-randomized studies included were evaluated for sources of bias using the NOS. All studies achieved an excellent score of 7/8 on the NOS. They attained maximum points for the ‘selection’ category. Some risk of bias was present due to heterogeneity of the population that reduced the comparability of the study cohorts.

**Table 2:** Newcastle-Ottawa Quality Assessment Scale for Cohort Study

**Author (year)**	**Selection**	**Comparability**	**Outcome**
Donlon 2021	***	**	**
Hoepner *et al.*	****	**	**
Lorenzen *et al.*	***	**	***
Misra *et al.*	***	**	**
Luc *et al.*	***	*	**
Port *et al.*	****	**	**
Lim *et al.*	***	*	**
Peixoto *et al.*	****	**	**
Bradley *et al.*	****	**	**
Mesenas *et al.*	****	**	**
Ott *et al.*	***	**	***
Kamarajah *et al.*	****	**	***

### Study and patient characteristics

Twenty-nine different studies were included in this paper.^3^^,^^11^^,^^14–31^ Eighteen of these studies were RCTs and the remainder were retrospective cohort studies. In total, this accounted for 5,026 patients. Patients underwent a combination of open, minimally invasive or hybrid procedures that included esophagectomy for esophageal tumors or gastrectomy junctional tumors. Studies included had adenocarcinoma histology. In all studies, there was a spread of pathological T and N stages of tumors (Table 1).

### Impact of oncological therapy on pCR rates

**Fig. 2:**
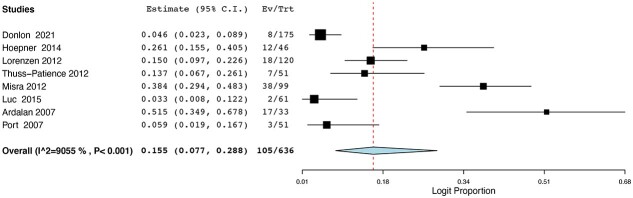
pCR rates in FLOT-based chemotherapy regimes.

**Fig. 3:**
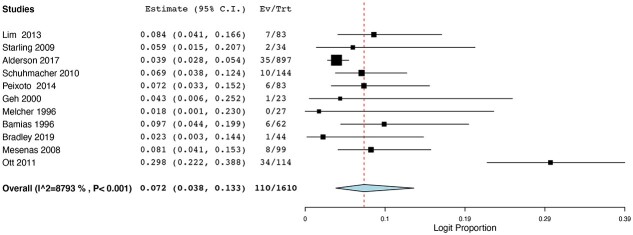
pCR rates in non FLOT-based chemotherapy regimes.

**Fig. 4:**
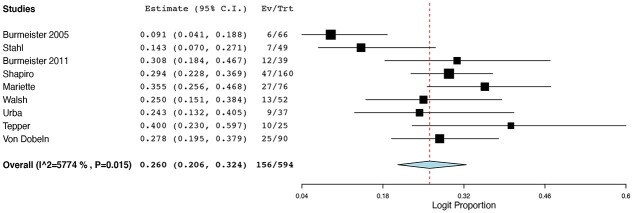
pCR rates in neoadjuvant chemoradiotherapy regimes.

Eight of the studies investigated the use of FLOT-based chemotherapy regimens, including FLOT or DCF. Eleven of the remaining studies used a non-FLOT-based protocol, usually ECF or ECX. Nine studies had investigated the outcomes following chemoradiotherapy. In total, 1,004 patients had undergone FLOT or DCF; 1,688 patients had received ECF or ECX and 1,022 patients had undergone chemoradiotherapy. The pCR rates ranged from 3.3% to 54% for FLOT regimes, while pCR rates were between 0% and 31% for ECF/ECX protocols. pCR rates ranged from 9.09% to 40% for the CRT cohorts. Pooled binary random-effects meta-meta-analysis of proportions showed a statistically significant higher incidence of pCR in FLOT-based chemotherapy at 0.148 (95%CI: 0.080, 0.259) (Fig. 2) compared with non-FLOT-based chemotherapy at 0.074 (95%CI: 0.042, 0.129) (Fig. 3). However, of the three cohorts, the pCR rates for the CRT groups were the highest at 0.250 (95%CI: 0.202, 0.306) (Fig. 4).

## DISCUSSION

This study highlights the different patterns in tumor regression of OAC after NAC, specifically between regimens with and without taxane-based agents (FLOT). Overall, the pCR rate was significantly higher for regimens such as FLOT and DCF that include taxanes, compared with conventional regimens such as ECF or ECX, but lower than that seen with neoadjuvant chemoradiotherapy. This finding is relevant in the current proposed transition toward an organ preservation strategy for esophageal cancer with a greater reliance on systemic therapies.

NAC aims to reduce the size of the tumor to lend higher R0 resection rates and decrease local and systemic recurrence, all of which have translated to better survival outcomes compared with historical figures when surgery was the primary sole treatment option. However, following the CROSS trial, the use of neoadjuvant CRT became more prevalent in the neoadjuvant setting due to better outcomes than NAC. For example, the combination of paclitaxel, carboplatin and 41.4 Gy/23 fractions resulted in a pCR of 29%; median OS of 49 months and 47% 5-year OS in 366 patients (75% adenocarcinoma). In comparison, the multi-centered RTOG 8911/Intergroup 0113 RCT (54% EAC) showed no survival advantage from pre- and postoperative 5-Fluorouracil and cisplatin compared with just surgery. The pCR rates and OS were also similarly lower than CRT in both the OEO2 and OEO5 trials. In their meta-analysis of surgery with NAC or CRT, Gebski *et al.*^32^ reported a 7% 2-year survival advantage for pre-operative chemotherapy and a 13% 2-year advantage for chemoradiotherapy. Similarly, the study by Stahel *et al.* showed a higher pCR in the CRT group compared with chemotherapy (17% vs. 2.5%), which was also associated with longer OS (3-year survival 43% vs. 27%).^33^ Hence, in most centers, the use of CRT is prevalent to downstage the disease, achieve tumor regression and permit surgical intervention.

Compared with conventional NAC and CRT, our study is in concordance with previous work that highlights the superiority of taxane-based NAC regimens. For example, the multi-centered FLOT4 RCT compared ECF/ECX (3 cycles pre- and postoperatively) with docetaxel, oxaliplatin, 5-FU and leucovorin (FLOT) in 300 patients, and demonstrated a pCR of 16% for the FLOT regimen compared with just 6% for ECF/ECX.^10^ Not only was this the highest pCR reported with chemotherapy alone, but also it recorded a major pathological response of 37% with FLOT. The use of taxanes to improve pCR is especially useful in the management of OACs, which are typically resistant to chemoradiotherapy. This difference in tumor biology is highlighted in the stratified analysis of the CROSS cohort, where pCR rates were 49% and 23% in ESCC and EAC, respectively. Naturally, this has led to ongoing RCTs, namely the ESOPEC, Neo-AEGIS and TOPGEAR trials as well as several phase II studies (NeoSCOPE, PROTECT 1402 and POWERRANGER) to optimize the components of current regimens and compare it against NACRT.^22^^,^^34–37^

### Future work: applicability of findings

Given that over 70% of patients who undergo an esophagectomy procedure have long-term symptoms that drastically reduces their quality of life, there is a clear push to solely rely on systemic therapies to manage EACs. The efficacy of FLOT in achieving pCRs adds credence to this idea and justifies the basis for the ongoing SANO trial.^38^^,^^39^ Briefly, the SANO trial is investigating the safety and efficacy of an organ-sparing approach in by randomizing patients with complete clinical regression (i.e. after neoadjuvant chemoradiotherapy no evidence of residual disease in two consecutive clinical response evaluations) to either active surveillance or standard oesophagectomy. In patients who achieve clinically complete response (cCR), the combination of regular surveillance imaging and endoscopies to monitor patients post-NAC/CRT means surgery can be offered if any recurrent or residual tumor is identified. Indeed, this is reliant on our ability to correlate cCR with pCR, which is the gold standard for assessing tumor response to systemic therapies. Future work can be aimed at using available technologies, including radiomics and CT scans, PET scans, endoscopy with biopsies, circulating tumor DNA (ctDNA) and mutational analyses, to evaluate clinical tumor response and build a criteria that correlates cCR with pCR to determine whether a patient can undergo surveillance or will instead require surgical intervention.^40^

### Limitations

Our review summarizes the existing work surrounding the use of NAC on tumor response; however, there are several limitations to this study. First, there are few studies that have directly compared the efficacy of various chemotherapy regimens in patients with OAC and reported the pCR, hence we could not generate an accurate comparative model but have identified ongoing studies aimed at this. Furthermore, pathological response is assessed using Tumor Regression Grading, which is typically either the Mandard or AJCC classification. Although both systems have good correlation, this still introduces a degree of heterogeneity that we could not account for. Third, most studies have reported OS outcomes, but other measures such as disease-free, recurrence-free and progression-free survival were not always reported. Similarly, a major drawback of both CRT and FLOT-based chemotherapy are their side effect profile. While a few studies reported them, pooled analysis could not be performed to directly compare their side effect profiles.

## CONCLUSION

Esophageal adenocarcinomas are malignancies associated with a prognosis and are challenging to manage, given their poor response to previously available systemic therapies. The use of enhanced FLOT-based regimens have improved the pCR rates, which have translated to better OS outcomes. Ongoing work is being carried out to directly compare these regimens and raises the possibility of avoiding surgical intervention if adequate tumor regression is achieved. Further work should be aimed at harnessing available technologies and investigations to accurately characterize clinical response to NAC, and determine whether an organ preservation strategy with surveillance could be employed for treating esophageal adenocarcinoma.
